# Contraceptive use in Latin America and the Caribbean with a focus on long-acting reversible contraceptives: prevalence and inequalities in 23 countries

**DOI:** 10.1016/S2214-109X(18)30481-9

**Published:** 2019-01-22

**Authors:** Rodolfo Gomez Ponce de Leon, Fernanda Ewerling, Suzanne Jacob Serruya, Mariangela F Silveira, Antonio Sanhueza, Ali Moazzam, Francisco Becerra-Posada, Carolina V N Coll, Franciele Hellwig, Cesar G Victora, Aluisio J D Barros

**Affiliations:** a Latin American Center for Perinatology/Women's Health and Reproductive Health of the Pan American Health Organization (CLAP/WR-PAHO/WHO), Montevideo, Uruguay; b International Center for Equity in Health (ICEH), Federal University of Pelotas, Pelotas, RS, Brazil; c Faculty of Medicine, Federal University of Pelotas, Pelotas, RS, Brazil; d Pan American Health Organization (PAHO), Washington, DC, USA; e Department of Reproductive Health and Research (RHR), World Health Organization, Geneva, Switzerland

## Abstract

**Background:**

The rise in contraceptive use has largely been driven by short-acting methods of contraception, despite the high effectiveness of long-acting reversible contraceptives. Several countries in Latin America and the Caribbean have made important progress increasing the use of modern contraceptives, but important inequalities remain. We assessed the prevalence and demand for modern contraceptive use in Latin America and the Caribbean with data from national health surveys.

**Methods:**

Our data sources included demographic and health surveys, multiple indicator cluster surveys, and reproductive health surveys carried out since 2004 in 23 countries of Latin America and the Caribbean. Analyses were based on sexually active women aged 15–49 years irrespective of marital status, except in Argentina and Brazil, where analyses were restricted to women who were married or in a union. We calculated contraceptive prevalence and demand for family planning satisfied. Contraceptive prevalence was defined as the percentage of sexually active women aged 15–49 years who (or whose partners) were using a contraceptive method at the time of the survey. Demand for family planning satisfied was defined as the proportion of women in need of contraception who were using a contraceptive method at the time of the survey. We separated survey data for modern contraceptive use by type of contraception used (long-acting, short-acting, or permanent). We also stratified survey data by wealth, area of residence, education, ethnicity, age, and a combination of wealth and area of residence. Wealth-related absolute and relative inequalities were estimated both for contraceptive prevalence and demand for family planning satisfied.

**Findings:**

We report on surveys from 23 countries in Latin America and the Caribbean, analysing a sample of 212 573 women. The lowest modern contraceptive prevalence was observed in Haiti (31·3%) and Bolivia (34·6%); inequalities were wide in Bolivia, but almost non-existent in Haiti. Brazil, Colombia, Costa Rica, Cuba, and Paraguay had over 70% of modern contraceptive prevalence with low absolute inequalities. Use of long-acting reversible contraceptives was below 10% in 17 of the 23 countries. Only Cuba, Colombia, Mexico, Ecuador, Paraguay, and Trinidad and Tobago had more than 10% of women adopting long-acting contraceptive methods. Mexico was the only country in which long-acting contraceptive methods were more frequently used than short-acting methods. Young women aged 15–17 years, indigenous women, those in lower wealth quintiles, those living in rural areas, and those without education showed particularly low use of long-acting reversible contraceptives.

**Interpretation:**

Long-acting reversible contraceptives are seldom used in Latin America and the Caribbean. Because of their high effectiveness, convenience, and ease of continuation, availability of long-acting reversible contraceptives should be expanded and their use promoted, including among young and nulliparous women. In addition to suitable family planning services, information and counselling should be provided to women on a personal basis.

**Funding:**

Wellcome Trust, Pan American Health Organization.

## Introduction

To achieve the goal of not leaving anyone behind, measurement of social inequalities is essential. Health inequities are related to social determinants, and Latin America and the Caribbean are still characterised by wide income and social inequalities, despite having made progress.^1^ In contrast with the Millennium Development Goals, health inequalities are central to the Sustainable Development Goals (SDGs), which recommend that assessment of health interventions at national level should be accompanied by stratified analyses to explore inequalities across subgroups.

Several countries in Latin America and the Caribbean have made important progress increasing the use of modern contraceptives, but important inequalities remain between and within countries.^2^, ^3^, ^4^, ^5^, ^6^, ^7^, ^8^ A study in ten countries covering the period from 1992 to 2012 has shown that the rise in contraceptive use was largely driven by an increased use of short-acting reversible contraceptives (SARCs).^7^ Long-acting reversible contraceptives (LARCs) are infrequently used, but they have several advantages over other types of modern contraceptives. LARCs (including intrauterine devices and hormonal implants) are safe, highly effective, independent of user compliance after insertion, cost-effective, and oestrogen free.^6^, ^9^, ^10^ An analysis of 43 countries^9^ showed that the lowest failure rates among contraceptive methods (other than permanent contraception) were observed for intrauterine devices and implants.

**Research in context**

**Evidence before this study**

Several countries in Latin America and the Caribbean have made important progress in increasing the use of modern contraceptives, but important inequalities remain between and within countries. Although long-acting reversible contraceptives—including intrauterine devices and hormonal implants—have several advantages over other types of modern contraceptives, a study analysing data from ten countries in Latin America and the Caribbean covering the period from 1992 to 2012 showed that the rise in contraceptive use was largely driven by short-acting methods.

**Added value of this study**

We analysed a sample population of 353 803 women from national health surveys of 23 countries in Latin America and the Caribbean, representative of 91% of all women of reproductive age in this region. We report the prevalence of modern contraceptive use among women at the time of the survey, and the proportion of women in need of contraception who were using a modern contraceptive method at the time of the survey, with a focus on inequalities in use prevalence of long-acting reversible contraceptives. We build on available academic literature by analysing a larger number of countries than previous studies and by disaggregating the data to identify priority groups for intervention.

**Implications of all the available evidence**

Our results show that contraceptive use varies widely across the region, and that in most countries short-acting reversible contraceptives are used much more frequently than long-acting reversible contraceptives. Many women in Latin America and the Caribbean would benefit from using long-acting reversible contraceptives, because of their high effectiveness, convenience, ease of continuation, and suitability for most women, including young and nulliparous women. Availability of long-acting reversible contraceptives should be expanded, along with information on their advantages and disadvantages. Clear and balanced information on a range of modern contraceptive methods should be offered to women, enabling them to make informed choices about their fertility.

In Latin America and the Caribbean, only 6·4% of women use an intrauterine device (7·7% in the Caribbean, 9·5% in Central America, 4·8% in South America).^11^ Availability of intrauterine devices is generally highest in the private sector and is limited in the public sector where the most vulnerable women receive care. Additionally, postpartum insertion of intrauterine devices is not universally practised in Latin America and the Caribbean. A multicountry study^8^ in this region highlighted socioeconomic inequalities in the types of modern contraceptives being used. In five of the six countries studied (Bolivia, Colombia, Dominican Republic, Guyana, and Peru—but not in Haiti), higher socioeconomic position was associated with more frequent use of permanent contraception and LARC. However, the study did not analyse the use of these two types of contraception separately. Generally, women who opt for permanent contraception might be quite distinct from those who choose LARC in terms of age or socioeconomic position. Therefore, separating the two methods might help us better understand these population subgroups.

In this study, we assessed contraceptive prevalence and demand for family planning satisfied among women 15–49 years of age in Latin America and the Caribbean, with a special interest in LARCs. We used data from national health surveys for the analyses. Between-country and within-country inequalities in the indicators were explored by stratifying the population samples by wealth quintiles, area of residence, age, education, ethnicity, and by a combined classification of wealth and area of residence. We built on available academic literature by analysing a larger number of countries than in previous studies, and disaggregating the data to identify subgroups with low contraceptive use in each country.

## Methods

### Data sources

Our data sources included demographic and health surveys, multiple indicator cluster surveys, and reproductive health surveys carried out since 2004. These are publicly available, nationally representative cross-sectional surveys with information on women of reproductive age. The three types of survey collect comparable reproductive, maternal, and child health indicators; more details on their design are available elsewhere.^12^, ^13^, ^14^ For Brazil, data were extracted from the 2013 national health survey, also a nationally representative survey that includes some reproductive indicators but is mainly focused on risk factors for chronic diseases.^15^ All surveys used multistage sampling procedures to select women aged 15–49 years for interview.

Analyses were based on sexually active women irrespective of marital status, with two exceptions: in Argentina and Brazil, the samples were restricted to women who were married or in a union, because no data were gathered on sexual activity in the month before the survey. For all other countries, women were considered sexually active if they were married or reported having had intercourse in the month preceding the interview.

According to the classification of contraceptives by Hubacher and Trussel,^16^ we categorised contraceptive methods as traditional or modern. Traditional contraceptive methods included rhythm methods (calendar, standard days, basal body temperature, symptothermal, and TwoDay), withdrawal method, lactational amenorrhoea method, and other traditional methods. Modern contraceptive methods were divided into three subcategories: long-acting reversible contraceptives (intrauterine devices and subdermal implants), short-acting reversible contraceptives (oral contraceptive pills, injectables, diaphragms and cervical caps, vaginal rings, male and female condoms, spermicidal agents, patch contraception, and emergency contraception), and permanent contraceptive methods (male and female sterilisation). Despite the 2015 WHO recommendations on classification of modern contraceptives^17^ we chose to use Hubacher and Trussel's classification, as did the authors of recent publications such as the 2015 UN report on trends in contraception use worldwide.^11^ The SDG indicator 3.7.1 (proportion of women of reproductive age who have their need for family planning satisfied with modern methods) also uses Hubacher and Trussel's classification (except for lactational amenorrhoea, which is considered modern).

This study did not require ethics approval. All analyses relied on publicly available databases that had removed all identifying data to guarantee participant anonymity. The institutions and national agencies in each country obtained ethics approval for the surveys.

### Procedures

Two sets of outcomes were analysed: contraceptive prevalence and demand for family planning satisfied (DFPS), calculated for all contraceptive methods and for modern methods only. Contraceptive prevalence was defined as the percentage of sexually active women aged 15–49 years who (or whose partners) were using a contraceptive method at the time of the survey. DFPS was defined as the proportion of women in need of contraception who were using a contraceptive method at the time of the survey. Women in need of contraception were defined as those who were fecund and did not want to become pregnant within the next 2 years or were unsure if or when they wanted to become pregnant. Pregnant women with a mistimed or unplanned pregnancy were also considered in need of contraception. and were included in the DFPS analysis. When data for contraceptive use were missing, women were considered as not using any contraception. Five surveys (Argentina, Brazil, Ecuador, Nicaragua, and Paraguay) did not include all variables needed to estimate DFPS. Given that DFPS and contraceptive prevalence are highly correlated, we estimated DFPS using the following predictive equation based on a recent analysis of 197 multiple indicator cluster surveys and demographic and health surveys:^18^

$$\text{logit}(DEPS)=0.61+0.68\log(CPR)+3.57CPR^{2}$$

### Statistical analysis

Survey data were stratified by household wealth quintiles, on the basis of the wealth index provided with the surveys. For reproductive health surveys, the wealth index was calculated using principal component analysis of household assets and building characteristics, following the methodology used by demographic and health surveys to calculate the wealth index.^19^ Adjustments were made for the area of residence (urban or rural) using linear regression models. The predicted values from the models constituted the adjusted wealth index that was divided into quintiles, with Q1 representing the poorest and Q5 the wealthiest 20% of all households.^19^, ^20^, ^21^ Information on wealth was not available for Cuba.

Additional stratifiers included area of residence (defined by the local census bureaus as urban or rural), education (none, any primary, and any secondary or higher), age (15–17 years, 18–19 years, and 20–49 years), ethnicity, and a combined classification of wealth and area of residence that consisted of ten categories. Analyses were stratified by ethnicity if the surveys provided self-reported information on ethnic group affiliation, language, or skin colour (see appendix for the variables available in each country). Three broad categories were used for ethnicity in our analyses: indigenous, afro-descendant, or reference if the participants had not self-assigned to indigenous or afro-descendant. Individuals not self-assigned to indigenous or afro-descendant groups were European descendants and those of mixed ancestry, and were used as the reference group in the analyses.^22^

For both indicators studied, absolute wealth-related inequalities were assessed with the slope index of inequality and relative wealth-related inequalities were assessed with the concentration index. The slope index of inequality is measured with a logistic regression model, to calculate the difference in percentage points between the fitted values of prevalence for the top and the bottom of the wealth distribution.^21^ The concentration index is measured by ranking individuals by their socioeconomic position (from poorest to richest) and plotting them against the cumulative proportion of health. If health is equally distributed across individuals, the concentration index would be zero. A positive concentration index indicates contraceptive use is found less often among poorer people and a negative concentration index indicates contraceptive use is found more often among poorer people. ^21^

All analyses were done in Stata (StataCorp, College Station, TX, USA; version 13.1) and adjusted for the sample design, including sample weights, clusters, and strata.

### Role of the funding source

This report contains the collective views of an international group of experts and does not necessarily represent the decisions or the stated policy of the Wellcome Trust, WHO, and the Pan American Health Organization. The funders of the study had no role in study design, data collection, data analysis, data interpretation, or writing of the report. The corresponding author had full access to all the data in the study and had final responsibility for the decision to submit for publication.

## Results

**Figure 1:**
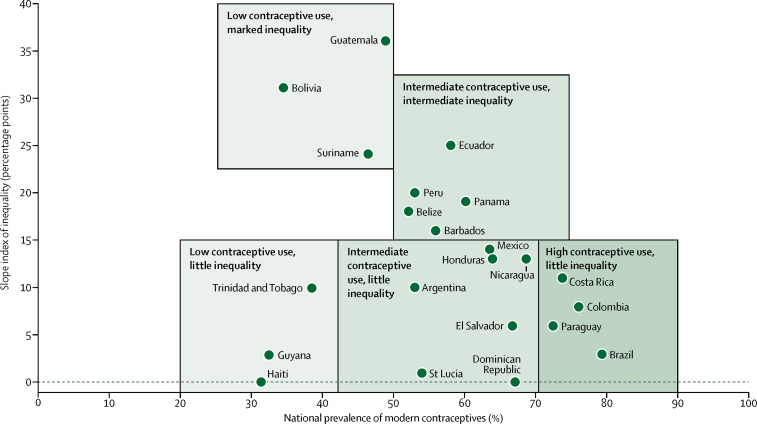
Wealth-related inequalities in modern contraceptive coverage Absolute inequality was measured with the slope index of inequality, which expresses the difference in percentage points between fitted values of modern contraceptive prevalence for the top and the bottom of the wealth distribution.

212 573 women from six demographic and health surveys, 13 multiple indicator cluster surveys, three reproductive health surveys, and one national health survey across 23 countries were included in our analyses (table 1). The median sample size was 7833 women per country. On average, contraceptive prevalence with any method (modern or traditional) was 6·4 percentage points higher than contraceptive prevalence with modern methods, indicating limited reliance on traditional methods in the region across Latin America and the Caribbean. Bolivia and Peru stood out with more than 20% of women using traditional contraceptive methods. DFPS with modern methods was greater than 80% in the following ten countries: Brazil, Paraguay, Cuba, Nicaragua, Costa Rica, Colombia, El Salvador, Dominican Republic, Mexico, and Ecuador (listed from highest to lowest DFPS). In Bolivia and Haiti, DFPS with modern methods was below 50%. We measured wealth-related inequalities in modern contraceptive coverage at national level (figure 1) and identified groups of countries with similar characteristics. Bolivia, Suriname, and Guatemala had low contraceptive prevalence and high inequality. Haiti, Guyana, and Trinidad and Tobago had low contraceptive prevalence and low inequality. Paraguay, Costa Rica, Colombia, and Brazil had high contraceptive prevalence and low inequality. Cuba had the third highest prevalence of modern contraceptives (74·9%), but information on wealth was not available.

**Table 1:** Contraceptive prevalence and demand for family planning satisfied based on the most recent national health surveys in Latin America and the Caribbean

	**Survey year**	**Survey type**	**CPR with any contraceptive method (%)**	**CPR with modern contraceptive methods (%)**	**DFPS with any contraceptive method (%)**	**DFPS with modern contraceptive methods (%)**	Number of women*
Argentina†	2011	MICS	55·4	53·0	78·6	76·5	21 660
Barbados	2012	MICS	59·6	56·0	75·1	70·9	1080
Belize	2011	MICS	55·3	52·2	75·4	71·4	2711
Bolivia	2008	DHS	61·6	34·6	75·5	42·4	10 847
Brazil*	2013	NHS	82·0	79·4	94·7	93·7	12 437
Colombia	2015	DHS	81·3	76·2	91·4	85·6	24 351
Costa Rica	2011	MICS	75·2	73·9	88·2	86·8	3428
Cuba	2014	MICS	76·2	74·9	90·3	89·5	7360
Dominican Republic	2014	MICS	68·5	67·1	84·2	82·9	19 883
Ecuador	2004	RHS	72·5	58·2	90·6	81·0	5654
El Salvador	2014	MICS	71·1	66·8	87·3	83·2	7833
Guatemala	2014	DHS	60·9	49·0	81·3	65·4	15 695
Guyana	2014	MICS	33·7	32·4	53·6	51·6	3848
Haiti	2012	DHS	34·8	31·3	48·5	43·7	8750
Honduras	2011	DHS	73·5	64·0	87·2	75·9	14 115
Mexico	2015	MICS	65·5	63·6	84·3	82·9	8148
Nicaragua	2006	RHS	72·8	68·8	90·8	88·5	9877
Panama	2013	MICS	62·9	60·3	77·3	74·5	6702
Paraguay	2008	RHS	84·2	72·6	95·4	90·7	4790
Peru	2012	DHS	76·6	53·1	90·7	62·0	15 753
St Lucia	2012	MICS	57·2	54·0	76·0	72·3	833
Suriname	2010	MICS	46·9	46·5	69·7	69·2	4324
Trinidad and Tobago	2006	MICS	42·9	38·5	61·1	56·3	2494

Contraceptive prevalence is the percentage of sexually active women aged 15–49 years who (or whose partner) were using a contraceptive method at the time of the survey. Demand for family planning satisfied is the proportion of women in need of contraception who were using a contraceptive method at the time of the survey. Demand for family planning satisfied for the surveys in Argentina, Brazil, Ecuador, Nicaragua, and Paraguay was estimated from contraceptive prevalence with a prediction equation.^18^ SEs for estimates of contraceptive prevalence and demand for family planning satisfied are in the appendix. MICS=multiple indicator cluster survey. CPR=contraceptive prevalence. DFPS=demand for family planning satisfied. DHS=demographic and health survey. NHS=national health survey. RHS=reproductive health survey.

* Estimates based on women who are married or in a union. All other estimates are based on women who are sexually active irrespective of marital status.

† Unweighted number of sexually active women analysed in each survey.

**Figure 2:**
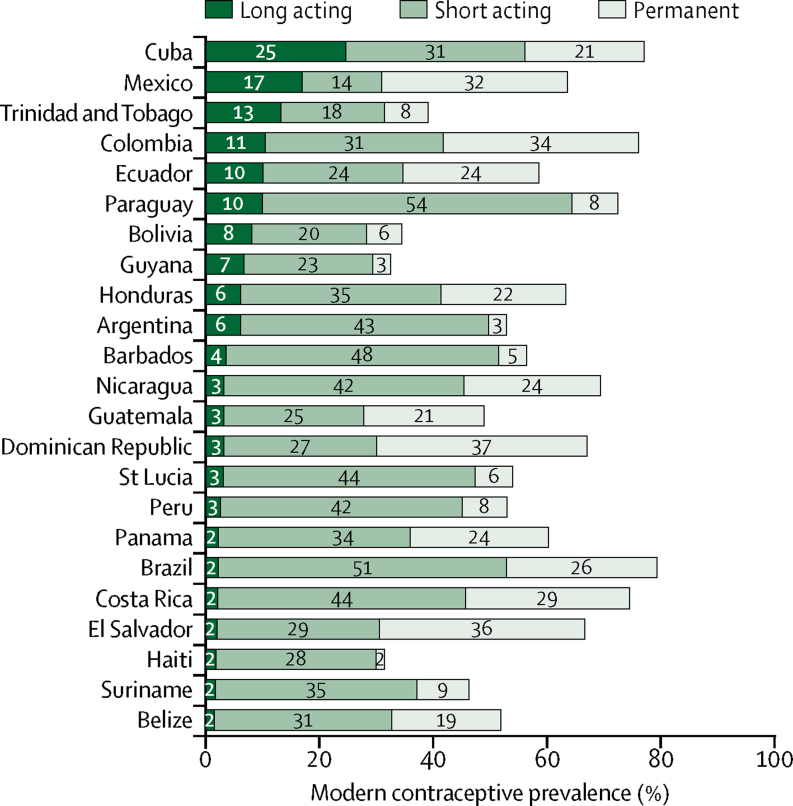
Modern contraceptive prevalence according to the type of contraceptive method being used (long acting, short acting, or permanent) among sexually active women by country Estimates for Argentina and Brazil are restricted to women married or in a union. All other estimates are based on sexually active women irrespective of marital status (see appendix for SEs of the estimates).

In most countries, SARCs were the most frequent type of contraceptive used by women (figure 2). 40% or more of women were using SARCs in Argentina, Barbados, Brazil, Costa Rica, Nicaragua, Paraguay, Peru, and St Lucia. By contrast, only Cuba, Colombia, Mexico, Ecuador, Paraguay, and Trinidad and Tobago had more than 10% of women adopting LARCs. The proportion of women (or their partners) relying on permanent contraception varied widely (ranging from 2% in Haiti to 37% in the Dominican Republic). Permanent contraception accounted for more than half of all methods being used in Mexico, Dominican Republic, and El Salvador. By contrast, less than 5% of women were relying on permanent contraception in Guyana, Argentina, and Haiti. Mexico had the lowest prevalence of SARCs (14%) and was the only country in which LARCs (17%) were more frequently used than SARCs. The most common method of contraception in this country was permanent contraception (32%).

**Figure 3:**
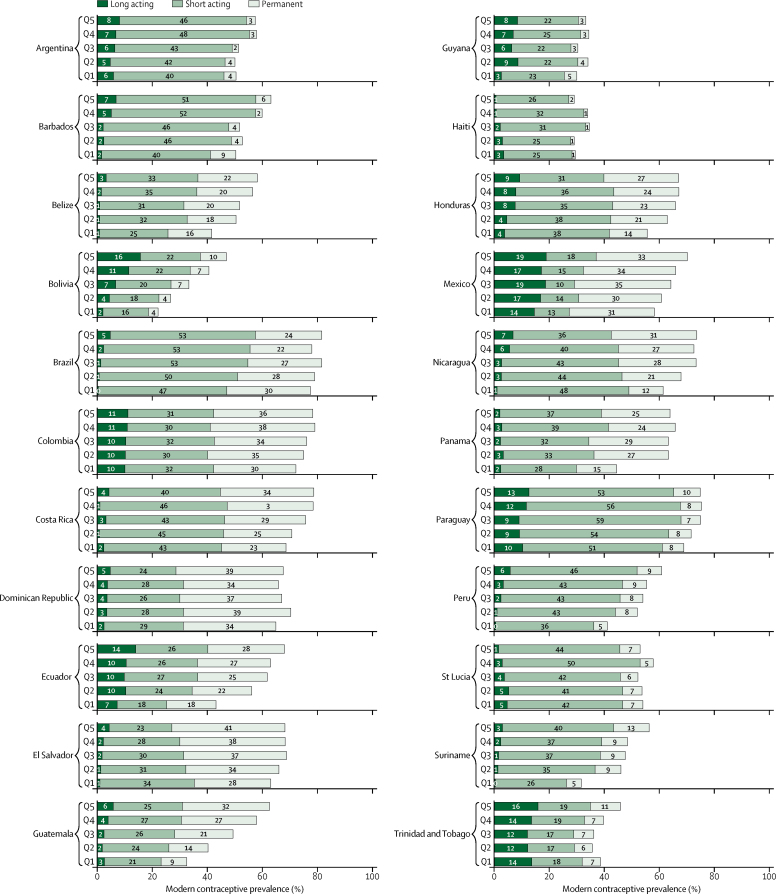
Modern contraceptive prevalence according to the type of contraceptive being used (long acting, short acting, or permanent) among sexually active women by country, stratified by wealth quintile Estimates for Argentina and Brazil are restricted to women married or in a union. All other estimates are based on sexually active women irrespective of marital status.

We assessed socioeconomic inequalities in the use of contraceptives in two ways. First, we explored the prevalence of modern contraceptives by wealth quintile (figure 3). Generally, countries did not show important variations according to wealth. However, in a few countries the use of LARCs was greater among richer women, particularly in Bolivia. In Haiti and St Lucia LARCs were used the most among the poorer quintiles. In Argentina, Colombia, and Panama the use of LARCs was similar for all women independent of wealth.

Second, we assessed the prevalence of LARCs according to wealth quintile, area of residence, age, education, ethnicity, and a combined classification of wealth and area of residence. Median LARC use was 4·2% in urban areas and 2·7% in rural areas and ranged from 2·5% in the poorest wealth quintile to 6·3% in the richest wealth quintile (table 2). For women with secondary education, median LARC use was higher (5·2%) than for those with primary education (2·8%) or no school education (2·2%) (see appendix for all stratified estimates). Median LARC use for indigenous women was 2·5%, whereas it was 4·2% for afro-descendant women and 3·8% for those in the reference group. Median use of LARCs among adolescents (1·1% in women aged 15–17 years and 2·0% in women aged 18–19 years) was lower than among women aged 20–49 years (3·8%). In conclusion, median LARC use was very low for all population subgroups, and socioeconomic differences were small in absolute terms. LARC use according to a combined classification of wealth and area of residence also showed no major differences between quintiles. Within each quintile, median LARC use was always slightly larger for women living in urban areas; the highest median prevalence of LARCs was 6·0% for urban women in the richest quintile, and the lowest was 2·3% for rural women in the poorest quintile.

**Table 2:** Prevalence and inequalities of LARCs among sexually active women according to wealth quintile and area of residence

	**Year**	**Prevalence of LARCs by wealth quintile (%)**					**Absolute inequality (SII, 95% CI)**	**Relative inequality (CIX, 95% CI)**	**Prevalence of LARCs by area of residence (%)**	
		Q1	Q2	Q3	Q4	Q5			Urban	Rural
Argentina*	2011	5·9%	4·7%	6·4%	6·8%	8·0%	3·2 (1·0 to 5·4)	9·4 (3·6 to 15·2)	6·4%	NA
Barbados	2012	1·6%	2·4%	2·2%	5·1%	6·8%	6·8 (1·9 to 11·7)	32·0 (13·8 to 50·2)	3·3%	4·7%
Belize	2011	0·8%	0·9%	0·9%	1·6%	3·2%	2·9 (0·7 to 5·1)	33·2 (13·5 to 53·0)	2·0%	1·1%
Bolivia	2008	2·1%	4·4%	6·7%	11·5%	15·7%	17·6 (15·0 to 20·1)	34·1 (30·2 to 38·0)	11·3%	3·5%
Brazil*	2013	0·5%	0·7%	1·3%	2·3%	4·7%	5·6 (3·3 to 7·9)	45·8 (35·0 to 56·6)	2·2%	1·0%
Colombia	2015	10·0%	10·2%	10·4%	10·9%	11·1%	1·4 (−1·5 to 4·4)	1·8 (−2·6 to 6·2)	10·6%	10·0%
Costa Rica	2011	2·4%	0·8%	3·1%	1·0%	4·2%	2·0 (−1·2 to 5·2)	20·9 (−2·1 to 43·8)	2·8%	1·7%
Cuba	2014	NA	NA	NA	NA	NA	NA	NA	25·1%	24·4%
Dominican Republic	2014	2·5%	3·5%	3·5%	3·7%	4·7%	2·2 (0·8 to 3·5)	10·3 (4·1 to 16·6)	4·1%	2·1%
Ecuador	2004	7·3%	10·4%	9·8%	10·5%	14·0%	6·5 (0·9 to 12·1)	10·9 (2·3 to 19·4)	11·6%	8·9%
El Salvador	2014	1·1%	1·2%	1·7%	2·5%	4·7%	4·2 (2·4 to 6·0)	33·4 (23·6 to 43·3)	2·8%	1·4%
Guatemala	2014	2·6%	1·8%	2·4%	3·9%	5·8%	4·2 (2·9 to 5·6)	20·5 (13·9 to 27·1)	4·3%	2·7%
Guyana	2014	2·5%	8·5%	6·3%	6·7%	8·4%	4·7 (0·9 to 8·6)	9·8 (0·4 to 19·2)	5·0%	7·3%
Haiti	2012	3·4%	2·9%	2·1%	0·7%	0·6%	−3·9 (−5·4 to −2·4)	−34·6 (−44·5 to −24·7)	0·9%	2·5%
Honduras	2011	3·7%	4·5%	7·6%	7·8%	9·2%	6·9 (5·0 to 8·8)	18·3 (14·2 to 22·5)	8·4%	4·9%
Mexico	2015	14·5%	16·6%	18·6%	17·0%	18·1%	3·6 (−5·3 to 12·5)	2·6 (−4·7 to 9·9)	17·4%	15·5%
Nicaragua	2006	1·0%	2·5%	2·6%	5·6%	6·8%	7·4 (5·5 to 9·3)	34·3 (28·6 to 40·0)	5·2%	1·6%
Panama	2013	2·3%	3·2%	2·3%	2·7%	2·0%	−0·6 (−2·5 to 1·3)	−1·6 (−14·9 to 11·8)	2·5%	2·5%
Paraguay	2008	10·3%	9·1%	9·0%	11·6%	12·6%	3·5 (−0·3 to 7·4)	6·2 (0·2 to 12·2)	11·3%	9·1%
Peru	2012	0·5%	1·0%	2·4%	3·2%	5·8%	6·6 (5·0 to 8·1)	40·8 (35·0 to 46·7)	3·3%	0·8%
St Lucia	2012	4·7%	5·2%	3·6%	2·8%	1·5%	−4·4 (−9·1 to 0·4)	−23·3 (−43·1 to −3·4)	3·1%	3·6%
Suriname	2010	0·4%	1·3%	1·5%	2·3%	2·9%	3·0 (1·2 to 4·7)	27·1 (13·7 to 40·6)	2·0%	1·0%
Trinidad and Tobago	2006	13·5%	12·1%	12·0%	13·5%	15·8%	3·0 (−2·0 to 8·0)	4·5 (−1·7 to 10·8)	NA	NA
Median (IQR)	..	2·5% (1·1–6·3)	3·4% (1·3–8·7)	3·3% (2·2–8·0)	4·5% (2·5–10·6)	6·3% (4·0–11·5)	3·6 (2·2–6·5)	14·6 (4·0–33·3)	4·2% (2·7–10·8)	2·7% (1·5–8·1)

SEs for the estimates of prevalence of long-acting reversible contraceptives according to wealth quintiles and area of residence are in the appendix. LARCs=long-acting reversible contraceptives. SII=slope index of inequality. CIX=concentration index.

* Estimates based on women who are married or in a union.

Few subgroups in our analyses showed LARC use greater than 20%. In Cuba, this was the case for all subgroups studied, but there was no information on wealth or ethnicity in the Cuban survey. In Mexico, LARC use was greater than 20% among adolescents. When we restricted our analyses to married Mexican adolescents using modern contraception, LARC accounted for 57·7% of all methods used.

## Discussion

We report on surveys from 23 countries in Latin America and the Caribbean, analysing a sample of 212 573 women to present the contraceptive prevalence and DFPS with modern methods. Taken together, the 23 countries comprise 91% of all women of reproductive age in the region.^23^

We found that modern contraceptive use and DFPS varied widely by country. In some countries modern contraceptive use was below 40%, and in others it was almost at 80%. In ten of the 23 countries DFPS was above 80%; of these ten countries, only Ecuador and Paraguay had a more substantial reliance on traditional methods. Peru had the highest share of traditional methods, with a difference of 23·5 percentage points between contraceptive prevalence with any method and with modern methods. In most countries, short-acting methods were most commonly used. Dominican Republic, El Salvador, and Colombia stood out because more than a third of sexually active women had been sterilised. In many countries in the region, LARCs were seldom used by any subgroup of the population.

The results presented mean that there is no single policy approach for Latin America and the Caribbean, because countries are in very different situations regarding family planning. Some countries also present large wealth-related inequalities in contraceptive use, notably Guatemala, Bolivia, Suriname, and Ecuador. In countries with low prevalence of contraceptives and low inequality, the challenge is to promote family planning with an equity approach so that the poorest women are not left behind. In countries with very high inequalities, it is essential to reach those who are already disadvantaged.

National LARC use among sexually active women was less than 5% in 13 of the 23 countries we studied. The only country in which LARCs were more frequently used than SARCs was Mexico. Countries with socioeconomic inequalities tended to have higher LARC use among richer women than poorer women. Although LARCs are suitable for women in nearly all situations, most contraceptive users were relying on SARCs or permanent methods.

The massive use of permanent contraception in some countries might indicate that women end up going for an irreversible alternative to avoid the limitations in access or difficulties in use related to other types of contraceptive methods. In Brazil, female sterilisation was the most prevalent contraceptive method in the 1990s, and a study^24^ suggested that many women opted for it to facilitate their entry into the workforce. LARCs could be a suitable alternative, given that they are highly effective, preserve women's rights to decide about their fertility in the future, and minimise the difficulties with access since regular restocking is not needed.

In many situations, the choice of contraceptive method is shaped by the health providers and their policies. In the 1980s, intrauterine devices were heavily promoted in several countries in Latin America and the Caribbean, and their use was common. With the HIV epidemic, condoms were strongly promoted because of their ability to prevent sexually transmitted diseases. And with lower doses of hormones, the contraceptive pill gained ground in many settings. We believe that countries should make available a wide mix of methods to health providers through their family planning programmes. With a choice of short-acting and long-acting contraceptives and the necessary information, women will then be able to make their informed choice, adopting the methods that best match their needs and culture.

LARCs are highly effective, economical, reversible, and not dependent on the woman or their partner taking any action (such as wearing a condom, taking a daily pill, or keeping tabs on fertile days). They do have disadvantages, notably the annulation of regular menstrual cycles in the case of implants, which might not be well accepted by some women. We believe that family planning programmes should give more attention to LARCs. The challenges to increase LARC uptake in Latin America and the Caribbean have been summarised by Bahamondes and colleagues.^25^ These challenges include barriers of a subjective nature (misinformation, myths, and beliefs) and of an objective nature (institutional, service-related, training-related, cost-related, and others). The barriers would have to be addressed to put LARCs in equal standing to other methods.

Our analyses have some limitations, notably the lack of data for some large countries in the region, such as Venezuela and Chile. Nevertheless, to our knowledge we have done the most comprehensive analysis so far in terms of the number of countries covered. We used indicators that include non-married sexually active women, and presented results for modern contraceptives and for any contraceptives. We reported contraceptive prevalence and demand for family planning satisfied. The data sources are reliable and account for more than 90% of all women of reproductive age in the region. Some surveys with smaller sample size have less precise estimates, but this should not bias the point prevalence estimate. For some countries the available information is already dated, with five of the 23 countries studied having data from before 2010, so the estimates might not reflect the current situation anymore. Our modern contraceptive definition is not in agreement with the latest WHO recommendation,^17^ but this choice is unlikely to have any effect on our results, since according to our data, the methods in disagreement with our definition are used by less than 2% of women in Latin America and the Caribbean. Wealth indices were used to assess economic status; such indices might vary according to the choice of assets, and they are affected by issues of comparability between urban and rural households.^26^ To address this issue, the asset indices used in the present analyses were adjusted to account for differences in asset importance in urban and rural areas.^27^

In conclusion, we found very different situations in terms of family planning across countries in Latin American and the Caribbean, with contraceptive use varying widely. There is also a varying degree of inequality across countries, favouring poor people when they exist. The percentage of women adopting permanent contraception is very high in a few countries, and in most of them SARCs are more commonly used than LARCs. We believe there is space to increase the uptake of LARCs in most countries, by including them in the mix of methods offered or by improving the information given to women about them. In any case, women should be the ones to decide on their reproduction, being able to make a free and informed choice on contraception.

For more on the **SDG indicator 3.7.1** see https://unstats.un.org/sdgs/metadata/files/Metadata-03-07-01.pdf
