# Supplementary material for: Contraceptive use in Latin America and the Caribbean with a focus on long-acting reversible contraceptives: prevalence and inequalities in 23 countries
Source: Lancet Glob Health. 2019 Jan 22;7(2):e227–35. doi: 10.1016/S2214-109X(18)30481-9 (PMC6367565; doi:10.1016/S2214-109X(18)30481-9)
Supplement: Supplementary appendix [file mmc1.pdf]

THE LANCET
Global Health

Supplementary appendix

This appendix formed part of the original submission and has been peer reviewed. We post it as supplied by the authors.

Supplement to: de Leon RGP, Ewerling F, Serruya SJ, et al. Contraceptive use in Latin America and the Caribbean with a focus on long-acting reversible contraceptives: prevalence and inequalities in 23 countries. *Lancet Glob Health* 2019; **7:** e227–35.

RESUMEN

Antecedentes

A pesar de la alta efectividad de los anticonceptivos reversibles de larga duración, el incremento en el uso de anticonceptivos se debe en gran parte a los métodos de corta duración. Varios países de América Latina y el Caribe han logrado avances importantes aumentando el uso de anticonceptivos modernos, pero persisten desigualdades importantes. Este estudio evaluó la prevalencia y demanda del uso de anticonceptivos modernos en América Latina y el Caribe utilizando los datos de las encuestas nacionales de salud.

Métodos

Se utilizaron datos de las encuestas de demografía y salud, encuestas de indicadores múltiples y encuestas de salud reproductiva realizadas desde 2004 hasta la fecha en 23 países de América Latina y del Caribe. Se analizaron los datos de mujeres entre 15 a 49 años, independientemente del estado civil, excepto en Argentina y Brasil, donde se utilizaron datos de mujeres casadas o en una unión. Se calculó el uso de contraceptivos y de la demanda de planificación familiar satisfecha. Se definió la prevalencia del uso de contraceptivos como el porcentaje de mujeres sexualmente activas (o su pareja) con edades entre 15 y 49 años que estaban usando un método anticonceptivo en el momento de la encuesta. Se definió la demanda de planificación familiar satisfecha como la proporción de mujeres fértiles que no deseaban quedar embarazadas y que usaban un método anticonceptivo en el momento de la encuesta. Los datos de la encuesta sobre el uso de anticonceptivos modernos fueron separados en tres grupos según el método de anticoncepción utilizado (de larga duración, corta duración o permanente). Además se estratificaron los datos de la encuesta por ingresos, área de residencia, educación, etnia, edad y una combinación ingresos-área de residencia. Se estimaron las desigualdades absolutas y relativas en el uso de anticonceptivos relacionadas con los ingresos.

Resultados

Fueron estudiadas un total de 212.573 mujeres de los 23 países incluidos. La prevalencia más baja del uso de anticonceptivos modernos se observó en Haití (31.3%) y Bolivia (34.6%). Las desigualdades fueron mayores en Bolivia, pero casi inexistentes en Haití. Por su parte, Brasil, Colombia, Costa Rica, Cuba y Paraguay presentaron una prevalencia de uso de anticonceptivos de más del 70% con menores desigualdades absolutas y relativas. En 17 de los 23 países el uso de anticonceptivos reversibles de larga duración fue inferior al 10%. Solo Cuba, México, Trinidad y Tobago, Colombia y Ecuador presentaron más del 10% de mujeres adoptando métodos anticonceptivos de larga duración. México fue el único país donde los métodos anticonceptivos de larga duración fueron utilizados con mayor frecuencia que los métodos de corta duración. Las mujeres menores de 19 años, mujeres indígenas, aquellas en los menores quintiles de riqueza, quienes viven en áreas rurales y las que no tienen educación mostraron un uso particularmente bajo de anticonceptivos reversibles de larga duración.

Interpretación

Los anticonceptivos reversibles de larga duración son poco utilizados en América Latina y el Caribe. Debido a su alta efectividad, conveniencia, facilidad de continuación, se debe ampliar la disponibilidad de este tipo de anticonceptivos y promover su uso, incluso en mujeres jóvenes y nulíparas. Esto significa que además de los programas de planificación familiar adecuados, se debe proporcionar información y asesoramiento a nivel personal, así como apoyo para las usuarias cuando presenten problemas con el método que está siendo utilizado.

RESUMO

Introdução

Apesar da alta efetividade dos métodos contraceptivos reversíveis de longa duração, o aumento do uso de contraceptivos tem sido amplamente impulsionado por métodos de curta duração. Diversos países da América Latina e Caribe apresentaram um aumento importante no uso de contraceptivos modernos, porém importantes desigualdades persistem. O objetivo deste estudo foi avaliar a prevalência de uso e a demanda por contraceptivos satisfeita com métodos modernos na região, utilizando dados de pesquisas nacionais de saúde.

Métodos

Foram utilizados dados do *Demographic and Health Survey* e *Multiple Indicator Cluster Survey* conduzidos a partir de 2004 em 23 países da América Latina e Caribe. As análises foram baseadas em mulheres de 15 a 49 anos, independentemente do estado civil, exceto na Argentina e no Brasil, onde as análises foram restritas a mulheres casadas ou em união. Foram estimadas a prevalência do uso de contraceptivos e a demanda por planejamento familiar satisfeita. Prevalência de uso de contraceptivos foi definida como o porcentual de mulheres sexualmente ativas com idade entre 15 e 49 anos que (ou cujo parceiro) estava utilizando algum método contraceptivo no momento da pesquisa. Demanda por planejamento familiar satisfeita foi definida como a proporção de mulheres em necessidade de contracepção que estava utilizando algum método contraceptivo moderno no momento da pesquisa. O uso de contraceptivos modernos foi calculado por tipo de método utilizado (contraceptivos de longa duração, curta duração ou permanente). Os resultados foram estratificados de acordo com riqueza, área de residência, educação, etnia, idade e uma combinação de riqueza e área de residência. Também foram estimadas desigualdades absolutas e relativas no uso, de acordo com riqueza.

Resultados

Foram analisadas 212.573 mulheres de 23 países. A menor prevalência de uso de contraceptivos modernos foi observada no Haiti (31,3%) e Bolívia (34,6%); a Bolívia apresentou alta desigualdade enquanto no Haiti praticamente não houve diferença entre os subgrupos. Brasil, Colômbia, Costa Rica, Cuba e Paraguai apresentaram prevalências de uso de contraceptivos modernos acima de 70%, com baixos níveis de desigualdades absolutas e relativas. O uso de contraceptivos reversíveis de longa duração foi inferior a 10% em 17 dos 23 países analisados. Apenas em Cuba, México, Trinidad e Tobago, Colômbia, Equador e Paraguai mais de 10% de mulheres adotaram algum destes métodos. O México foi o único país em que uso de métodos contraceptivos de longa duração foi superior ao uso dos de curta duração. Mulheres com menos de 19 anos, indígenas, mais pobres, que vivem em áreas rurais ou que não têm escolaridade apresentaram níveis ainda mais baixos de uso de contraceptivos reversíveis de longa duração.

Conclusões

Contraceptivos reversíveis de longa duração são pouco utilizados na América Latina e Caribe. Devido à sua alta efetividade, conveniência e facilidade de continuação do uso, a disponibilidade destes contraceptivos deve ser ampliada e seu uso promovido, inclusive entre mulheres jovens e nulíparas. Isso significa que, além de programas adequados de planejamento familiar, informação e aconselhamento de forma pessoal deve ser fornecido as mulheres, além de suporte às usuárias quando elas encontram problemas com o método utilizado.

Table S1. Classification of ethnicity (or proxy variable) used for each country.

Country<br>Year	Information	Group	Definition (as in original questionnaire)
Belize	Ethnicity	Reference	Mestizo, others
2011	(head of household)	Indigenous	Maya
MICS		Afro-descendant	Creole, Garifuna
Bolivia	Ethnicity	Reference	No ethnic affiliation declared
2008	(woman)	Indigenous	Quechua, Aymara, Guarani, other indigenous group
DHS		Afro-descendant	Not available
Brazil	Ethnicity/skin color	Reference	White
2006	(woman)	Indigenous	Indigenous
NHS		Afro-descendant	Brown or Black
Colombia	Ethnicity	Reference	Other
2015	(woman)	Indigenous	Native Colombian
DHS		Afro-descendant	Raizal from Archipelago, Palanquero from San Basilio, Black/Mulato/Afro-Colombian/Afro-descendant
Ecuador	Ethnicity/skin color	Reference	White, Mestizo
2004	(woman)	Indigenous	Indigenous
RHS		Afro-descendant	Black
Costa Rica	Ethnicity	Reference	Others
2011	(head of household)	Indigenous	Indigenous
MICS		Afro-descendant	Black / Afro-Costarricense
Guatemala	Ethnicity	Reference	Ladino/Mestizo
2014	(woman)	Indigenous	Maya, Xinca
DHS		Afro-descendant	Not available
Guyana	Ethnicity	Reference	Mixed race/East Indian
2014	(head of household)	Indigenous	Amerindian
MICS		Afro-descendant	African
Honduras	Ethnicity	Reference	No ethnic affiliation declared

2011	(woman)	Indigenous	Tolupan, Pech (Paya), Misquito, Nahoa, Lenca, Tawaka (Sumo), Maya Chorti
DHS		Afro-descendant	Garifuna, black English
Mexico	Ethnicity	Reference	Non-indigenous household
2015	(head of household)	Indigenous	Indigenous household
MICS		Afro-descendant	Not available
Nicaragua	Ethnicity	Reference	Mestizo from the Caribbean coast, no ethnicity declared
2006	(woman)	Indigenous	Rama, Mayangna-Sumu, Miskitu, Ulwa, Xiu-Sutiava, Nahoa-Nicarao, Chorotega-Nahua-Mange, Cacaopera-Matagalpa
RHS		Afro-descendant	Not available
Panama	Ethnicity	Reference	Others
2013	(head of household)	Indigenous	Indigenous
MICS		Afro-descendant	Black or afro-descendant
Paraguay	Idiom	Reference	Spanish or Spanish/guarani speakers
2008	(household)	Indigenous	Guarani only speakers
RHS		Afro-descendant	Not available
Peru	Idiom	Reference	Spanish speakers
2012	(household)	Indigenous	Quechua, Aymara, other indigenous
DHS		Afro-descendant	Not available
St Lucia	Ethnicity	Reference	Mixed descent, East Indian, other
2012	(head of household)	Indigenous	Not available
MICS		Afro-descent	
Suriname	Ethnicity	Reference	Creole/Indian/Javanese /Mixed race
2010	(head of household)	Indigenous	Indigenous/ Amerindian
MICS		Afro-descendant	Marron
Trinidad Tobago	Ethnicity	Reference	Indian, mixed, other
2006	(head of household)	Indigenous	
MICS		Afro-descendant	

Table S2. Prevalence (and standard errors) of LARC use among sexually active women according to wealth woman's age, education and ethnicity

		Age			Education			Ethnicity		
**Country**	**Year**	**15-17**	**18-19**	**20-49**	**None**	**Primary**	**Secondary**	**Indigenous**	**Afro**	**Reference**
Argentina[a]	2011	0.8 (0.4)	0.8 (0.4)	7.6 (0.4)	6.4 (4.4)	8.3 (0.9)	6.0 (0.3)	NA	NA	NA
Barbados	2012	0.0 (0)	2.9 (2.9)	3.9 (0.7)	NA	2.8 (2.8)	3.9 (0.7)	NA	NA	NA
Belize	2011	0.0 (0)	1.2 (1.2)	1.6 (0.3)	2.2 (1.7)	0.8 (0.3)	2.1 (0.4)	0.0 (0)	1.6 (0.5)	1.7 (0.4)
Bolivia	2008	1.9 (1)	3.4 (1)	8.7 (0.4)	2.2 (0.8)	5.4 (0.4)	12.6 (0.6)	6.3 (0.4)	NA	11.9 (0.6)
Brazil[a]	2013	NA	0.1 (0.1)	2.1 (0.3)	0.2 (0.2)	0.8 (0.2)	2.8 (0.4)	1.0 (0.2)	1.1 (0.7)	3.2 (0.5)
Colombia	2015	9.3 (1.3)	15.0 (1.4)	10.3 (0.4)	3.7 (1)	8.0 (0.5)	11.2 (0.5)	10.0 (1.3)	10.3 (1)	10.6 (0.4)
Costa Rica	2011	0.0 (0)	0.6 (0.5)	2.5 (0.5)	0.0 (0)	2.6 (0.8)	2.3 (0.6)	1.0 (0.9)	11.2 (8.8)	2.3 (0.5)
Cuba	2014	28.0 (5.9)	22.3 (4.6)	25.0 (1.1)	NA	22.5 (6.5)	25(1.1)	NA	NA	NA
Dominican Republic	2014	4.9 (1.2)	5.5 (0.9)	3.5 (0.2)	1.5 (0.8)	2.3 (0.3)	4.3 (0.3)	NA	NA	NA
Ecuador	2004	3.1 (1.5)	7.9 (2.2)	10.8 (0.6)	5.4 (1.7)	10.0 (1)	11.3 (0.8)	7.4 (1.6)	9.7 (2.4)	10.8 (0.7)
El Salvador	2014	0.5 (0.4)	0.7 (0.3)	2.2 (0.2)	0.1 (0.5)	1.0 (0.2)	3.1 (0.4)	NA	NA	NA
Guatemala	2014	2.1 (0.8)	2.0 (0.6)	3.5 (0.2)	1.8 (0.3)	2.3 (0.2)	5.9 (0.4)	2.7 (0.3)	NA	3.8 (0.3)
Guyana	2014	0.9 (0.9)	0.4 (0.4)	7.1 (0.7)	9.0 (3.6)	9.3 (1.8)	6.1 (0.6)	2.5 (1.1)	3.1 (0.8)	8.8 (1.1)

Haiti	2012	0.9 (0.9)	0.3 (0.2)	1.9 (0.2)	2.9 (0.5)	2.3 (0.3)	0.8 (0.2)	NA	NA	NA
Honduras	2011	2.1 (0.9)	3.5 (0.8)	7.1 (0.3)	2.7 (0.9)	5.5 (0.3)	9.1 (0.6)	8.2 (0.9)	10.0 (2.5)	6.4 (0.3)
Mexico	2015	21.9 (4.6)	24.6 (3.3)	16.9 (1.1)	7.5 (2.6)	13.0 (1.1)	18.5 (1.3)	17.2 (2.9)	NA	17.2 (1.1)
Nicaragua	2006	1.3 (0.5)	2.3 (0.8)	3.8 (0.3)	0.9 (0.7)	2.6 (0.4)	5.2 (0.5)	1.8 (1.3)	NA	3.8 (0.3)
Panama	2013	2.4 (2.4)	0.5 (0.2)	2.5 (0.4)	3.8 (2)	1.8 (0.4)	2.6 (0.4)	1.5 (0.3)	4.2 (1.4)	2.2 (0.4)
Paraguay	2008	0.8 (0.6)	3.7 (1.1)	11.3 (0.7)	12.5 (5.6)	8.8 (0.8)	11.7 (0.8)	8.1 (0.8)	NA	11.9 (0.8)
Peru	2012	0.0 (0)	0.1 (0.1)	2.8 (0.2)	0.2 (0.2)	1.4 (0.3)	3.2 (0.3)	0.7 (0.2)	NA	2.8 (0.2)
St Lucia	2012	0.0 (0)	0.0 (0)	3.8 (0.8)	0.0 (0)	4.1 (1.4)	3.3 (0.8)	NA	3.9 (0.9)	1.4 (1)
Suriname	2010	0.0 (0)	0.0 (0)	1.9 (0.3)	1.0 (0.7)	1.0 (0.3)	2.1 (0.3)	1.3 (0.9)	0.7 (0.3)	2.0 (0.3)
Trinidad and Tobago	2006	11.1 (6.1)	8.5 (3)	13.6 (0.7)	NA	10.2 (1.2)	14.4 (0.8)	NA	13.0 (1.1)	13.6 (0.9)
**Median**		**1.1**	**2.0**	**3.8**	**2.2**	**2.8**	**5.2**	**2.5**	**4.2**	**3.8**

[a] Estimates based only on women married or in a union

Table S3. Prevalence (and standard errors) of LARC use among sexually active women according to the combination of wealth and area of residence (urban/rural).

Country	Year	Wealth quintiles and area of residence									
		Q1 urban	Q1 rural	Q2 urban	Q2 rural	Q3 urban	Q3 rural	Q4 urban	Q4 rural	Q5 urban	Q5 rural
Argentina[a]	2011	5.9 (0.7)	NA	4.7 (0.5)	NA	6.4 (0.6)	NA	6.8 (0.7)	NA	8 (0.8)	NA
Barbados	2012	1.1 (0.7)	2.3 (2.2)	2.2 (1.2)	2.6 (1.8)	0 (0)	6.2 (2.8)	6 (2.4)	3.4 (2.1)	6 (2.4)	8.1 (3.1)
Belize	2011	0 (0)	1 (0.6)	0.8 (0.6)	1 (0.6)	0.6 (0.4)	1.1 (0.7)	1.9 (0.8)	1.1 (0.8)	4.2 (1.2)	1.5 (0.8)
Bolivia	2008	2.4 (1.8)	2.1 (0.3)	5.6 (1.3)	4 (0.7)	7.4 (0.8)	4.6 (0.9)	11.5 (0.8)	11.1 (2.6)	15.7 (1)	10.1 (6.2)
Brazil[a]	2013	0.6 (0.3)	0.2 (0.1)	0.7 (0.2)	0.8 (0.5)	1.2 (0.4)	2.4 (1.4)	2.2 (0.5)	4.6 (2.7)	4.8 (0.9)	1.1 (1.1)
Colombia	2015	9.4 (1.3)	10.1 (0.7)	10 (0.6)	10.7 (1.3)	10.5 (0.6)	2 (1.9)	10.9 (0.9)	7.5 (5.2)	11.1 (1.1)	2.8 (2.5)
Costa Rica	2011	1.6 (0.9)	2.7 (1.2)	1 (0.4)	0.7 (0.5)	3.8 (2.2)	2.2 (1)	1.4 (0.6)	0.1 (0.1)	4.7 (1.7)	2 (1)
Cuba	2014	NA	NA	NA	NA	NA	NA	NA	NA	NA	NA
Dominican Republic	2014	3.5 (0.5)	1.6 (0.3)	4 (0.6)	2.3 (0.7)	3.7 (0.5)	2.7 (0.6)	3.8 (0.6)	2.7 (0.9)	4.9 (0.6)	2.6 (1.2)
Ecuador	2004	10.5 (2.7)	6.8 (1.9)	10.4 (1.9)	10.4 (1.4)	9.2 (1.3)	10.9 (1.6)	10.8 (1.3)	8.8 (2.1)	14.6 (1.6)	8.9 (2.6)
El Salvador	2014	0.9 (0.7)	1 (0.3)	1.4 (0.5)	1.2 (0.3)	1.8 (0.5)	1.6 (0.5)	2.2 (0.7)	2.1 (1)	4.3 (0.8)	4.9 (2.6)
Guatemala	2014	3.9 (1.7)	2.4 (0.4)	1.6 (0.7)	1.8 (0.3)	2.8 (0.6)	2.3 (0.4)	3.6 (0.5)	4.4 (0.8)	6 (0.6)	4.6 (1.2)
Guyana	2014	5.7 (2.6)	1.9 (0.4)	4.7 (2.3)	9.6 (1.9)	3.9 (1.8)	6.9 (1.6)	3.6 (1.5)	8.7 (1.6)	6.9 (2.5)	9.2 (1.8)

Haiti	2012	NA	3.4 (0.6)	0.7 (0.8)	3 (0.6)	2.7 (0.6)	1.8 (0.5)	0.3 (0.1)	1.7 (1.2)	0.7 (0.2)	0 (0)
Honduras	2011	3.6 (1.8)	3.7 (0.4)	4.4 (1)	4.5 (0.5)	9.2 (1.1)	5.9 (0.7)	8 (0.9)	7 (1.3)	9.4 (0.8)	7.5 (1.6)
Mexico	2015	14.9 (2.2)	14.3 (1.5)	17 (2)	16.4 (2.7)	19.8 (1.9)	9.6 (2.1)	15.2 (1.6)	38.3 (12.7)	19.3 (4.4)	7.5 (3.6)
Nicaragua	2006	0.8 (0.8)	1 (0.2)	3.1 (0.9)	2.3 (0.4)	3.2 (0.7)	1.5 (0.5)	5.9 (0.9)	2.2 (1.5)	6.7 (0.8)	7.8 (3.2)
Panama	2013	0.9 (0.5)	2.6 (0.5)	4.2 (1.9)	2 (0.5)	2.2 (0.8)	2.5 (0.9)	2.7 (0.8)	2.2 (1.7)	1.7 (0.6)	3.9 (1.7)
Paraguay	2008	10.2 (2.7)	10.3 (1.3)	11 (1.8)	7 (1.4)	10.3 (1.6)	6 (1.3)	11.3 (1.4)	13.3 (2.5)	12.5 (1.5)	13.5 (4.1)
Peru	2012	0.4 (0.3)	0.5 (0.1)	0.8 (0.3)	1.1 (0.3)	2.5 (0.5)	1.4 (0.7)	3.3 (0.5)	0.5 (0.5)	5.8 (0.7)	0 (0)
St Lucia	2012	3.9 (2.7)	4.9 (2.3)	4.6 (2.5)	5.3 (2.3)	2.7 (1.9)	3.9 (1.8)	3.6 (2.4)	2.7 (1.5)	0 (0)	1.7 (1.2)
Suriname	2010	0 (0)	0.5 (0.2)	1.4 (0.6)	1.2 (0.5)	1.4 (0.6)	1.8 (0.8)	2.6 (0.7)	1.1 (0.6)	3.1 (0.8)	1.6 (0.9)
Trinidad and Tobago	2006	NA	NA	NA	NA	NA	NA	NA	NA	NA	NA
**Median**		**3.4**	**2.3**	**4.0**	**2.3**	**3.2**	**2.6**	**3.6**	**3.1**	**6.0**	**4.3**

[a] Estimates based only on women married or in a union

Table S4. Standard errors of the estimates of contraceptive prevalence (CPR) and demand for family planning satisfied (DFPS) at country level, type of contraception being used (long-acting, short-acting and permanent contraception) and of the LARC use by wealth quintiles and area of residence. Analyses based on sexually active women unless otherwise stated.

**Country**	**ISO code**	**Year**	**CPR (any method)**	**CPR (modern methods)**	**DFPS (any method)**	**DFPS (modern methods)**	**Long-acting**	**Short-acting**	**Permanent**	**Long-acting reversible contraception (LARC)**						
										**Wealth quintiles**					**Area**	
										Q1	Q2	Q3	Q4	Q5	Urban	Rural
Argentina[a]	ARG	2011	0.8	0.8	NA[b]	NA[b]	0.3	0.8	0.2	0.7	0.5	0.6	0.7	0.8	0.3	NA
Barbados	BRB	2012	1.7	1.8	1.8	1.9	0.7	1.7	0.7	1.0	1.0	1.1	1.8	1.8	0.8	1.2
Belize	BLZ	2011	1.2	1.3	1.0	1.0	0.3	1.1	0.9	0.5	0.4	0.4	0.6	0.6	0.5	0.3
Bolivia	BOL	2008	0.7	0.6	0.6	0.7	0.4	0.5	0.3	0.3	0.6	0.7	0.8	1.0	0.5	0.4
Brazil[a]	BRA	2013	0.6	0.6	NA[b]	NA[b]	0.2	0.8	0.8	0.2	0.2	0.4	0.5	0.9	0.3	0.4
Colombia	COL	2015	0.5	0.5	0.3	0.4	0.4	0.5	0.6	0.6	0.6	0.6	0.9	1.1	0.5	0.6
Costa Rica	CRI	2011	1.6	1.6	1.1	1.2	0.4	1.4	1.8	0.9	0.3	1.3	0.4	0.4	0.7	0.4
Cuba	CUB	2014	1.2	1.2	0.9	0.9	1.1	1.1	1.0	NA	NA	NA	NA	NA	1.3	2.0
Dominican Republic	DOM	2014	0.6	0.6	0.4	0.4	0.2	0.5	0.6	0.3	0.5	0.4	0.5	0.5	0.3	0.3
Ecuador	ECU	2004	0.8	1.0	NA[b]	NA[b]	0.6	0.8	0.8	0.2	0.4	0.5	0.9	0.8	0.5	0.2

El Salvador	SLV	2014	0.7	0.8	0.5	0.6	0.2	0.7	0.8	0.3	0.3	0.3	0.6	0.6	0.3	0.2
Guatemala	GTM	2014	0.6	0.7	0.5	0.7	0.2	0.5	0.5	0.4	0.3	0.4	0.4	0.5	0.3	0.2
Guyana	GUY	2014	1.1	1.1	1.5	1.5	0.6	1.0	0.4	0.6	1.6	1.3	1.1	1.1	1.2	0.7
Haiti	HTI	2012	0.9	0.9	1.0	1.0	0.2	0.8	0.2	0.6	0.6	0.4	0.3	0.2	0.2	0.3
Honduras	HND	2011	0.5	0.5	0.4	0.5	0.3	0.5	0.5	0.4	0.4	0.7	0.7	0.7	0.5	0.3
Mexico	MEX	2015	1.2	1.3	0.9	0.9	1.1	0.8	1.1	1.3	1.6	1.7	2.0	2.0	1.3	1.7
Nicaragua	NIC	2006	0.6	0.7	NA[b]	NA[b]	0.3	0.7	0.6	0.2	0.4	0.5	0.9	0.8	0.5	0.2
Panama	PAN	2013	1.3	1.3	1.2	1.3	0.4	1.2	1.0	0.5	1.0	0.7	0.8	0.8	0.5	0.3
Paraguay	PRY	2008	0.6	0.8	NA[b]	NA[b]	0.6	0.9	0.5	1.2	1.2	1.2	1.3	1.4	0.9	0.8
Peru	PER	2012	0.5	0.6	0.3	0.7	0.2	0.6	0.3	0.1	0.2	0.5	0.5	0.7	0.3	0.1
St Lucia	LCA	2012	2.0	2.0	1.9	1.9	0.8	1.8	1.1	1.9	1.9	1.5	1.3	1.3	1.1	0.9
Suriname	SUR	2010	1.0	1.0	1.2	1.2	0.2	0.8	0.6	0.2	0.4	0.5	0.6	0.6	0.3	0.2
Trinidad and Tobago	TTO	2006	1.1	1.1	1.3	1.3	0.7	0.8	0.6	1.6	1.4	1.4	1.5	1.5	NA	NA

[a] Estimates based on women married or in a union. All other estimates are based on women sexually active irrespective of marital status.

[b] DFPS was estimated from CPR using a prediction equation [18].
